# Collapsing Focal Segmental Glomerulosclerosis and Acute Kidney Injury Associated With Chimeric Antigen Receptor T-Cell (CAR-T) Therapy: A Case Report

**DOI:** 10.1016/j.xkme.2021.06.011

**Published:** 2021-08-09

**Authors:** Ratna Acharya, Biljana Horn, Xu Zeng, Kiran Upadhyay

**Affiliations:** 1 Divisions of General Pediatrics, University of Florida, Gainesville, FL; 2 Pediatric Hematology-Oncology, University of Florida, Gainesville, FL; 3 Department of Pediatrics, Division of Anatomic Pathology, Department of Pathology, University of Florida, Gainesville, FL; 4 Division of Pediatric Nephrology, Department of Pediatrics, University of Florida, Gainesville, FL

**Keywords:** Chimeric antigen receptor T cells, collapsing FSGS, cytokine release syndrome, acute kidney injury, leukemia

## Abstract

Chimeric antigen receptor T (CAR-T) cell treatment is a rapidly emerging therapy for relapsed/refractory hematologic malignancies. Although cytokine release syndrome is a common complication, a concomitant development of biopsy-proven collapsing glomerulopathy and acute kidney injury (AKI) has not been described with CAR-T cell therapy. We report a man in his early 20s with relapsed/refractory pre–B-cell acute lymphoblastic leukemia and compensated liver cirrhosis who received 3 courses of CD19-directed CAR-T cells. After the third CAR-T cell therapy, he developed severe cytokine release syndrome accompanied by new onset of nephrotic syndrome and AKI. Cytokine release syndrome was treated with tocilizumab. His kidney biopsy showed collapsing glomerulopathy, glomerulitis, and interstitial nephritis along with complete podocyte foot-process effacement. Due to disease progression, he was subsequently treated with bispecific CD19-directed CD3 T-cell engager antibody, blinatumomab, during which he developed another episode of cytokine release syndrome with exacerbation of nephrotic-range proteinuria and his AKI progressed to stage 3 chronic kidney disease. Excess cytokine-induced podocyte and renal tubulointerstitial injury and/or “on-target off-tumor” direct renal cell toxicity are the probable mechanisms of kidney injury. Further such reports will increase our understanding of the pathophysiologic basis of kidney injury with CAR-T treatment.

## Introduction

CD19-specific chimeric antigen receptor T (CAR-T) cells can induce long-term remission in refractory or relapsed B-cell acute lymphoblastic leukemia (ALL) in children and young adults.^1^ The CAR-T cells recognize the specific surface antigen on the malignant cells such as CD19 and become highly activated to kill these cells.^1^

Cytokine release syndrome is a common occurrence with CAR-T cell therapy.^2^ Acute kidney injury (AKI) is uncommon but can occur due to cytokine-induced injury or as a result of an “on-target, off-tumor” phenomenon.^3^^,^^4^ However, to our knowledge, biopsy-proven collapsing glomerulopathy as a cause of nephrotic syndrome in association with AKI and subsequent chronic kidney disease has not been reported. We report such an association in a patient with relapsed B-cell ALL who received CD19 CAR-T cell therapy.

## Case Report

A Hispanic man in his early 20s had precursor B-cell ALL diagnosed at 17 years of age. During his initial chemotherapy, nonalcoholic liver cirrhosis was documented on liver biopsy, which prompted a reduction in chemotherapy. Eight months after completion of chemotherapy, his B-cell ALL relapsed with blasts expressing CD19, CD20, and CD22 antigens. He did not achieve remission with re-induction chemotherapy and was not eligible for bone marrow transplantation due to liver cirrhosis, so we proceeded with CAR-T cell therapy. Three doses of CAR-T cells were generated, each one containing 1.5 ×10^8^ (1.5 ×10^6^ per kg) autologous CD19 CAR-T cells (tisagenlecleucel [Kymriah]; Novartis Pharmaceuticals).

The patient received 2 CAR-T cell infusions preceded by lymphodepleting chemotherapy with fludarabine and cyclophosphamide. The second infusion was administered 2.5 months after the first one due to loss of B-cell aplasia. He did not receive any steroids during the 2 initial CAR-T cell treatments. He tolerated the first and second infusions well without cytokine release syndrome, neurotoxicity, or other complications.

Two months after the second infusion, relapse was diagnosed (16% blasts that were still CD19 positive). In discussion with CAR-T experts and due to lack of other treatment options, he received the third CAR-T infusion 5 months after the first infusion. Four days postinfusion, he developed grade 2 cytokine release syndrome, manifested by fever, hypotension, tachycardia, and hypoxia with profound elevation in serum interleukin 6 (IL-6; peak, >1,500 pg/mL; normal, <6 pg/mL), C-reactive protein (peak, 65.12 mg/L), prothrombin time and international normalized ratio (peak, 15.8 seconds and 1.4), partial thromboplastin time (peak, 114 seconds), ferritin (peak, 21,028 ng/mL), and D-dimer (>20 μg/mL) values. Soluble IL-2 receptor α was not measured. There was pancytopenia, hypertriglyceridemia, and evidence of mild tumor lysis syndrome, but no neurotoxicity. He was managed with intravenous hydration, antibiotics, and allopurinol. Two doses of 800 mg of tocilizumab intravenously were administered on day 6 after the third CAR-T therapy, with resolution of clinical signs and marked improvement in levels of the inflammatory markers of cytokine release syndrome within a week after tocilizumab therapy. Corticosteroids were not used.

A week after the third CAR-T therapy, the patient developed new-onset nephrotic-range proteinuria with random urinary protein-creatinine ratio reaching up to 60 mg/mg (normal, <0.2 mg/mg). There was no hematuria. There was oliguria, anasarca, and interval weight gain of 12 kg (13% of body weight). Serum albumin levels mostly varied between 1.5 and 2 g/dL. This was consistent with a new-onset nephrotic syndrome. Liver enzyme levels were normal despite the presence of nodular cirrhosis on a sonogram. There was no portal hypertension on a Doppler ultrasound. There was splenomegaly. An echocardiogram showed normal cardiac anatomy. A kidney sonogram showed right kidney of 12.2 cm and left kidney of 12.7 cm in length with increased echogenicity and no hydronephrosis. There was marked hypogammaglobulinemia requiring intravenous immunoglobulin infusions.

Serum protein electrophoresis showed marked hypoglobulinemia and hypoalbuminemia without any discrete spikes, including M spike. Urinary protein electrophoresis showed predominant albuminuria and absence of mono- or oligoclonal bands. Lupus serologic tests, HIV, parvovirus B19, and Epstein-Barr virus polymerase chain reaction, cryoglobulin, and hepatitis viral panel results were all negative. Nasopharyngeal severe acute respiratory syndrome coronavirus 2 (SARS-CoV-2) polymerase chain reaction was negative. Serum complement levels were normal. There was no history of herbal medication use. Nephrotic syndrome was managed with 25% albumin and furosemide infusions. Two weeks later, the patient's serum creatinine level started increasing from a baseline value of 0.52 to 0.75 mg/dL to a peak value of 2.02 mg/dL. The patient was not receiving other nephrotoxic agents.

**Figure 1:**
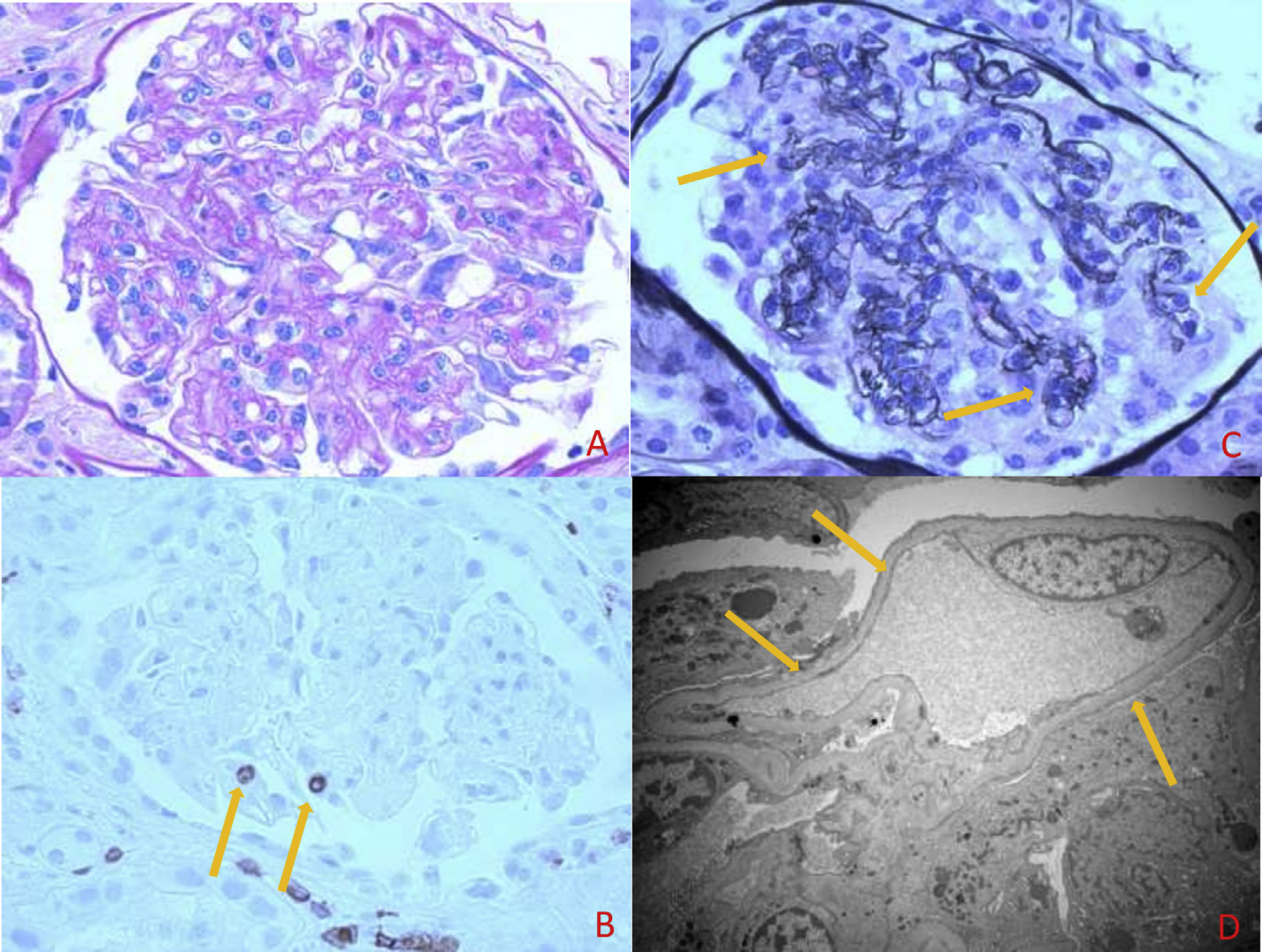
Kidney biopsy findings. (A) Light microscopy shows a glomerulus with relatively normal mesangial cellularity and mildly increased mesangial matrix (hematoxylin and eosin stain; original magnification, 40×10). (B) Immunohistochemical (IHC) stain for CD3 shows margination of CD3-positive T lymphocytes in the glomerulus. Interstitial inflammatory infiltrate also composed of CD3-positive T lymphocytes. (IHC; original magnification, 40×10). (C) Global collapse of glomerular capillary loops and podocyte hyperplasia in 1 glomerulus (silver stain; original magnification, 40×10). (D) Electron microscopy shows complete podocyte foot-process effacement (original magnification, ×5,000).

Kidney biopsy showed 17 glomeruli with mild mesangial expansion, glomerular margination of CD3-positive T lymphocytes, and global collapse of glomerular capillary loops with podocyte hyperplasia in 3 glomeruli (Fig 1A-C). There was no tubular microcysts formation. There was mild diffuse interstitial inflammation, consisting of mainly CD3-positive lymphocytes and rare CD20-positive lymphocytes. Electron microscopy showed complete podocyte foot-process effacement (Fig 1D). Bone marrow evaluation showed 66% lymphoblasts expressing CD19 antigen.

As a salvage therapy, the patient received a 28-day course of a bispecific CD19-directed CD3 T-cell engager antibody construct (blinatumomab [Blincyto]; Amgen) that was started 6 weeks after the third CAR-T cell infusion This was again complicated by 2 more episodes of grade 2 cytokine release syndrome, requiring 3 additional doses of tocilizumab. Nephrotic syndrome persisted with mean random urinary protein-creatinine ratio of 40 mg/mg, but edema had resolved with diuresis. His serum creatinine level peaked at 2.91 mg/dL (estimated glomerular filtration rate, 27 mL/min/1.73 m^2^). Dialysis was not required. Due to persistent disease and absence of CD19 expression after 28 days of blinatumomab treatment, no further plans for targeted therapy were made. He was discharged home with a serum creatinine level of 2.0 mg/dL on treatment with spironolactone, losartan, statin, lactulose, vitamin K, and ursodiol. Eleven weeks after the third CAR-T cell therapy, he was started on a palliative chemotherapy consisting of a short course of oral steroid (40 mg twice daily for 10 days), vincristine, and 6-mercaptopurine. At the most recent outpatient follow-up visit 13 weeks after the third CAR-T infusion, serum creatinine and albumin levels were 2.10 mg/dL (eGFR, 42 mL/min/1.73 m^2^), and 3.8 g/dL, respectively. There was persistent but decreasing proteinuria with a random urinary protein-creatinine ratio of 6.5 mg/mg.

## Discussion

Common complications following CD19 CAR-T therapy are cytokine release syndrome, neurologic toxicities, infections, tumor lysis syndrome, and prolonged cytopenias.^5^ Besides eliminating CD19 leukemia cells, these redistributed T cells proliferate and release excess proinflammatory cytokines, leading to local organ damage by activating tissue-resident immune cells.^6^ The most common manifestations of cytokine release syndrome are fever, hypotension, pulmonary and hepatic dysfunction, third spacing, serosal exudates, and bone marrow suppression.^6^ The risk for and severity of cytokine release syndrome increase with greater disease burden and higher CAR-T dose.^7^ Less commonly, AKI can occur secondary to cytokine-induced vasodilation, decreased cardiac output, increased vascular permeability, and third spacing of fluids leading to intravascular volume depletion.^3^

In a study of 75 pediatric patients and young adults with refractory/relapsed B-cell ALL who received a single dose of tisagenlecleucel, cytokine release syndrome developed in 77% and 8% had AKI, fluid overload developed in 5%, and dialysis was required in 9% of patients, all within 8 weeks after CAR-T infusion.^8^ Similarly, in a study of tisagenlecleucel in adult patients with relapsed or refractory diffuse large B-cell lymphoma, cytokine release syndrome occurred in 58% but dialysis was required in only 5% of patients.^9^

Gupta et al^10^ reviewed 78 adults receiving CAR-T therapy for diffuse large B-cell lymphoma; AKI occurred in 19%. In a retrospective review of 46 adult patients with non-Hodgkin lymphoma, the cumulative incidence rate of AKI was 30% at day 100 after CAR-T therapy.^11^ Lee et al^12^ described a 50-year-old patient with diffuse large B-cell lymphoma with nephrotic-range proteinuria and nonoliguric AKI who developed cytokine release syndrome along with clinical and laboratory findings of hemophagocytic lymphohistiocytosis a week after CAR-T therapy. However, none of the patients in these studies had a kidney biopsy and hence the exact kidney pathophysiology was unknown.

Our report is unique because a renal histology of collapsing glomerulopathy along with AKI was described in association with CAR-T therapy for the first time. Although the patient also had clinical features of hemophagocytic lymphohistiocytosis, in the absence of serum soluble IL-2 receptor α and natural killer cell activity testing, we could not definitely establish the diagnosis of hemophagocytic lymphohistiocytosis. Hence, this report emphasizes the possibility of secondary hemophagocytic lymphohistiocytosis being on the spectrum of severe cytokine release syndrome in patients who have received CAR-T therapy. Podocyte injury secondary to abnormal T-cell activation and/or high proinflammatory cytokine levels leading to collapsing focal segmental glomerulosclerosis has been described in association with hemophagocytic lymphohistiocytosis.^13^ Also, given the temporal relationship between the third CAR-T therapy and development of proteinuria, nephrotic syndrome is most likely a manifestation of cytokine release syndrome secondary to CAR-T therapy; however, paraneoplastic nephrotic syndrome as a manifestation of refractory B-cell leukemia cannot be ruled out.

The podocyte is a vital component of the glomerular filtration barrier, the injury of which leads to passage of albumin in Bowman space causing nephrotic syndrome.^14^ Podocyte foot-process effacement is the hallmark of podocyte injury, as observed in our patient. Podocyte injury in association with CAR-T therapy could be due to cytokine release syndrome. Idiopathic nephrotic syndrome is widely considered to be a T-cell dysfunction. Elevated serum and urine levels of various T-cell–derived circulating factors or cytokines, including IL-6, have been described in idiopathic nephrotic syndrome during relapse.^15^^,^^16^ Cytokines may induce podocyte damage by causing increased sulfation of glomerular basement membrane, thereby altering the charge selectivity of the membrane, and increased catabolism of the glomerular basement membrane heparan sulfate, leading to a reduction in the negative charge.^17^

The cytokine hypothesis is supported by the clinical response of the proteinuria with steroids and calcineurin inhibitors, an immediate relapse of nephrotic syndrome following kidney transplantation in some patients, and occurrence of albuminuria in rats infused with lymphocytes from patients with nephrotic syndrome.^18^ In our case, steroid was not used as either a premedication for CAR-T cell infusion or as a treatment of cytokine release syndrome or nephrotic syndrome to permit CAR-T cell expansion to treat the refractory leukemia. However, steroids may be beneficial in preventing and/or treating cytokine release syndrome either alone or in conjunction with tocilizumab.^3^ In addition to being an approved therapy for severe CAR-T cell–induced cytokine release syndrome, tocilizumab also has been described to induce remission of nephrotic syndrome secondary to AA amyloidosis in patients with rheumatoid arthritis and systemic juvenile idiopathic arthritis.^19^^,^^20^ With regard to glomerulitis and interstitial nephritis, IL-6, a proinflammatory cytokine, has been shown to be expressed in the inflamed glomeruli and tubulointerstitial cells, leading to glomerular and tubulointerstitial injury.^21^ Our patient had extremely elevated serum IL-6 levels, which could have contributed to the glomerular and interstitial injury.

Other potential mechanisms of kidney injury with CAR-T therapy are crosstalk between the infiltrated glomerular and interstitial CAR-T lymphocytes with the renal tubular epithelial cells and podocytes and an “on-target off-tumor” phenomenon.^22^^,^^23^ The crosstalk occurs when the infiltrated interstitial and glomerular lymphocytes produce cytokines, which then activate the renal tubular epithelial cells and podocytes.^22^ In turn, tubular cells and podocytes potentially produce a variety of inflammatory mediators that further amplify the local inflammatory response. Podocytes have been shown to produce cytokines such as tumor necrosis factor α, IL-6, and IL-8 at relatively low levels under basal conditions; however, in response to inflammation, they produce significantly increased levels of these cytokines.^24^ Another probable mechanism of kidney injury is an on-target off-tumor phenomenon in which the reactivated T cells against tumor antigens may cross-react with the off-target renal cells due to the possible presence of shared target antigen such as CD19.^4^ However, it remains to be studied whether podocytes or renal tubular cells express CD19.

Risk-adapted dosing with lower CAR-T cell doses in patients with high marrow blast counts and use of tocilizumab and corticosteroids for the treatment of cytokine release syndrome may reduce or ameliorate the renal toxicity without significantly compromising the efficacy. Also, early screening for proteinuria in patients who have sustained post–CAR-T AKI may aid in the early diagnosis of nephrotic syndrome.

Limitations of this report include unavailability of CAR-T cell, CD19, and IL-6 staining in the kidney biopsy specimen, unavailability of serum levels of other cytokines and soluble IL-2 receptor α, and absence of podocyte gene mutation panel including APOL-1 testing for a possible second-hit phenomenon. Also, the potential roles of plasmapheresis and rituximab in CAR-T–associated nephrotic syndrome need to be examined in future studies.
